# Feeling the Beat: Bouncing Synchronization to Vibrotactile Music in Hearing and Early Deaf People

**DOI:** 10.3389/fnins.2017.00507

**Published:** 2017-09-12

**Authors:** Pauline Tranchant, Martha M. Shiell, Marcello Giordano, Alexis Nadeau, Isabelle Peretz, Robert J. Zatorre

**Affiliations:** ^1^Faculty of Psychology, University of Montreal Montreal, QC, Canada; ^2^International Laboratory for Brain, Music, and Sound Montreal, QC, Canada; ^3^Centre for Interdisciplinary Research on Music, Media, and Technology Montreal, QC, Canada; ^4^Centre for Research on Brain, Language, and Music Montreal, QC, Canada; ^5^Montreal Neurological Institute, McGill University Montreal, QC, Canada; ^6^Input Devices and Music Interaction Lab, McGill University Montreal, QC, Canada

**Keywords:** dancing, beat sychronization, vibrotactile, deafness, sensorimotor integration

## Abstract

The ability to dance relies on the ability to synchronize movements to a perceived musical beat. Typically, beat synchronization is studied with auditory stimuli. However, in many typical social dancing situations, music can also be perceived as vibrations when objects that generate sounds also generate vibrations. This vibrotactile musical perception is of particular relevance for deaf people, who rely on non-auditory sensory information for dancing. In the present study, we investigated beat synchronization to vibrotactile electronic dance music in hearing and deaf people. We tested seven deaf and 14 hearing individuals on their ability to bounce in time with the tempo of vibrotactile stimuli (no sound) delivered through a vibrating platform. The corresponding auditory stimuli (no vibrations) were used in an additional condition in the hearing group. We collected movement data using a camera-based motion capture system and subjected it to a phase-locking analysis to assess synchronization quality. The vast majority of participants were able to precisely time their bounces to the vibrations, with no difference in performance between the two groups. In addition, we found higher performance for the auditory condition compared to the vibrotactile condition in the hearing group. Our results thus show that accurate tactile-motor synchronization in a dance-like context occurs regardless of auditory experience, though auditory-motor synchronization is of superior quality.

## Introduction

Dancing is a widespread human activity, shared universally across cultures and throughout human history (Nettl, 2000). At the basis of this activity is the ability to synchronize body movements to a musical beat, a behavior known as beat synchronization. Since music is typically experienced through the auditory modality, much research on beat synchronization has focused on synchronization to sounds (for a review, see Repp and Su, 2013). However, music can also be experienced through the tactile sense: Sound waves can generate vibrations in nearby objects, and these vibrations can be sensed by the somatosensory system via mechanoreceptors in the body. This experience is typical in a social dance setting, such as in a night club, where it is common for individuals to feel the music's bass as vibrations through the floor and/or resonating inside the chest cavity. These low-frequencies are also where the most salient beat information is generally transmitted (Van Dyck et al., 2013), and as such, the vibrations generated by these frequencies may be particularly useful for beat synchronization.

Although the experience of music is often accopanied by tactile vibrations, whether or not these vibrations are relevant for guiding beat synchronization while dancing under certain circumstances remains unknown. Recent research indicates that people are capable of synchronizing movements to vibrations outside of a dancing context. For example, trained guitarists are able to synchronize playing a guitar melody to a vibrotactile click-track that is administered with an actuator attached to the upper arm. In this case, guitar-plucks were performed as accurately when synchronizing to vibrations as to an auditory click-track (Giordano and Wanderley, 2015). Non-musicians are also able to tap along to vibrotactile beats administered to the fingertip (Brochard et al., 2008; Elliott et al., 2010) or along the back (Ammirante et al., 2016), although for more complex rhythms the somatosensory modality may be less effective than the auditory modality for beat delivery (Ammirante et al., 2016). While these studies show that some vibrotactile beat synchronization is possible in certain contexts, it remains unclear whether or not this behavior is relevant for dance.

For a hearing person in a dancing context, vibrotactile beat information from music is typically accompanied by auditory information as well. Presumably this auditory information can guide beat synchronization should the vibrations be insufficient, or interact with the vibrations in some way. However, for an individual with hearing loss, the vibrations generated by music may be the main sensation available to guide beat synchronization to the sound. Deaf people often participate in social dancing situations (Darrow, 1993), but little research has examined this behavior. In this population, music is typically experienced through vibrations, as evidenced by the development and effectiveness of vibrotactile platforms for musical experiences in the deaf (Karam et al., 2009; Baijal et al., 2012). In terms of beat synchronization, one recent study demonstrated that early deaf people are able to synchronize a hand-tapping motion to a visual stimulus, and in fact, perform better than hearing people when the stimulus is discrete (a flashing dot) rather than continuous (a bouncing ball) (Iversen et al., 2015). Furthermore, when deaf individuals, but not hearing, synchronized with a continuous visual stimulus, their tapping patterns suggest that visual timing may access higher-order beat perception mechanisms.

This enhancement in using visual input in deaf people suggests that auditory deprivation may lead to compensatory enhancements in visuomotor coordination, a possibility that is in line with evidence that the visual system undergoes cross-modal plasticity after deafness. For example, the early deaf are better than the hearing at detecting visual motion (Shiell et al., 2014). In deaf cats, this enhancement is obliterated when a specific region of the auditory cortex is selectively deactivated, which demonstrates that cross-modal activity in the auditory cortex supports this behavior (Lomber et al., 2010). In terms of somatosensation, there is some behavioral evidence that deaf people are more sensitive to vibrotactile stimulation than hearing people (Levanen and Hamdorf, 2001), and that the auditory cortex is reorganized to process vibrotactile stimuli (Levanen et al., 1998; Auer et al., 2007). We propose that this potential cross-modal somato-auditory plasticity may translate into an enhanced ability to synchronize to vibrotactile stimuli.

The purpose of the current experiment was to test beat synchronization to vibrotactile stimulation in a dance context, in both deaf and hearing people. We built a vibrotactile display platform that mimicked the resonance of a wooden floor, and asked participants to perform a full-body bouncing motion in time to a musical track. This musical track was presented either through sound alone or via vibrations felt through the platform. Movements were recorded with a motion capture system and the quality of synchronization was assessed. Based on previous research on beat synchronization to vibrotactile stimulation in hearing individuals (Brochard et al., 2008; Giordano and Wanderley, 2015; Ammirante et al., 2016), we predicted that hearing people would be able to synchronize to vibrotactile stimulation as well as they do to auditory stimulation. Furthermore, we predicted that deaf people would be better than hearing people at synchronizing to vibrotactile stimulation, following the hypothesis that auditory deprivation leads to compensatory enhancement of other sensory modalities.

## Materials and methods

### Ethics statement

The experiment was carried out in accordance with the recommendations of the McGill University Research Ethics Board II. All participants gave written informed consent in accordance with the Declaration of Helsinki.

### Participants

Participants were recruited from the Montreal area and were compensated for their participation. During the recruitment process, participants were screened for dance and musical experience: Participants who reported a dislike of dancing or extensive training in dance or music were excluded. A total of 9 deaf individuals were recruited, however one was excluded during testing because of an inability to synchronize bouncing, which was identified visually by the experimenter during the practice portion of the experiment (see Procedure). One additional deaf and one hearing participant were excluded based on their poor synchronization abilities (see Results). The final participant sample included 14 hearing (10 females, 5 males; mean age: 35.2 years, range 21–53 years, *SD* = 10.5) and 7 deaf people (6 females, 2 males; mean age: 37.0 years, range 26–49 years, *SD* = 10.0). Groups were matched for number of years of education (deaf group: mean = 17.1; *SD* = 3.2; range = 13–20. Hearing group: mean = 15.8; *SD* = 2.5; range = 12–21).

From the deaf group, two participants reported hereditary congenital deafness, two reported congenital deafness of unknown etiology, one was deafened from septicaemia at birth, one from encephalitis at 8 months of age, and one's deafness was discovered around the age of 1 year old, with unknown etiology. All seven deaf participants reported profound deafness (>90 dB loss). All deaf participants had some experience using a hearing aid (either because they were required to wear it for school or because their parents introduced it), but had minimal hearing aid use after adolescence and no cochlear implant history. From the hearing group, all participants reported normal hearing.

All deaf participants used sign language as their primary language. Five deaf participants reported that their first language was langue des signes québécoise (LSQ). One deaf participant had delayed language acquisition and primarily used gestures prior to learning sign language in late childhood, and one participant reported a first language of spoken French and attended oral school for deaf children prior to learning sign language in late childhood.

### Stimuli

We composed a simple musical track (see Supplementary Materials) with Ableton Live software (http://www.ableton.com/en/live) to simulate instruments. This track consisted of a regular beat in a bass drum, with metric rhythms by percussion instruments (snare drum, cymbal, and clapper) and an intermittent three-note melody. This track was presented at either 110, 115, or 120 beats-per-minute (BPM) and lasted for 128 beats (~1 min of time). For the two vibrotactile conditions (with masking and with earmuffs, see below), we wanted to simulate the features of the sound that would be conveyed in an ecologically valid setting, such as in a dance club. Accordingly, we filtered the stimuli to produce a frequency response similar to a wooden floor (see Supplementary Materials). This filter was created by using an accelerometer to record the frequency responses from one of the floors at the Centre for Interdisciplinary Research on Music, Media and Technology at McGill University (Montreal, Canada) and from the custom-made vibrating platform used to present the stimuli (see apparatus below). The ratio of these two frequency responses was then used to filter the musical stimuli (Giordano, 2015).

### Equipment

Stimuli were presented in three different conditions: auditory (for the hearing group only), vibrotactile with masking (mask), and vibrotactile with earmuffs (muff). For the auditory condition, stimuli were delivered at a comfortable volume through two loudspeakers, positioned at 150 cm height, 186 cm away from the participant at 75 degree angles to the left and right.

Vibrotactile conditions were delivered via a custom-made vibrating platform (Giordano, 2015), consisting of a 60 × 60 cm plank of composite wood, raised 10 cm off the ground. A loudspeaker-like vibrating actuator (Clark Synthesis TS209 Tactile Transducer) was fastened to the center underside of the plank and connected to an amplifier. The vibrations of the platform generated a quiet noise (LAFmax = 35.9 dB). To ensure that participants did not hear this noise, in the muff condition the participants wore industrial grade earmuffs (noise reduction rating = 27 dB). To mitigate the effect of bone conduction of vibrations to the ear, in the mask condition the participants listened to brown noise through headphones (Sennheiser HD 280 Pro). The volume of the noise was adjusted for each participant if necessary to a level that was reported as comfortable and sufficient to block the sound of the platform vibrations. In both the muff and mask vibrotactile conditions, participants reported that they were unable to hear the vibrations of the platform.

We used a ten-camera motion capture system (Qualisys Oqus) to collect participant movement data. The cameras were calibrated before each testing session. Each camera used infrared reflections to detect the unique three-dimensional positions of 28 reflective markers attached to the participant's body, at a 100 Hz sampling rate. This data was synchronized with the stimuli sent to the platform and the speakers via a Qualisys Analog interface (USB-2533) and recorded with Qualisys Track Manager software (http://www.qualisys.com/), which reconstructed the motion of each marker in real time.

### Procedure

Participants were tested individually. Participants and experimenters wore protective eyeglasses as a safety precaution to protect the eyes from the infrared light emitted by the motion capture system. The experimenter explained the task verbally, and for deaf participants these instructions were translated by a sign language interpreter. Reflective markers were attached on the participant's body on the front and back of the hips (4 markers), front and back of the shoulders (4 markers), the elbows (2 markers), the top of the hands (2 markers), the front of the knees (2 markers), the heels (2 markers), the top surface of the foot near the toes on the inside and the outside (4 markers), and around the head (8 markers). Participants were instructed to perform a full-body vertical bouncing movement by bending their knees in time with a musical beat, which was either heard through the loudspeakers or felt through the vibrating platform. This movement was first described to the participants verbally without any visual demonstration. Participants were told to bend their knees in parallel, keeping their hips facing forward, their arms relaxed and resting at their sides, and their feet flat on the platform. Participants were asked to synchronize their bouncing motion to the tempo of the auditory or vibrotactile stimuli as accurately as possible, bouncing on every beat.

For testing, participants stood barefoot on the vibrotactile platform in the middle of the room, facing away from the experimenter. Before testing trials began, participants underwent a spontaneous condition where they were instructed to perform the bouncing motion at a constant rate in the absence of the stimuli for 45 s. The purpose of this spontaneous bouncing condition was to screen participants for a potential difficulty producing a regular movement. Participants then did a practice trial with examples of the testing stimuli and were given corrections from the experimenter if necessary. The level of the vibrations was adjusted if necessary to ensure that the sensory information was clear, and for hearing participants, that the vibrations were not audible with the earmuffs or the masking.

Each condition (music, muff, and mask) was presented in same-condition blocks of six trials. In each block, stimuli at each tempo (110, 115, 120 BPM) were presented twice in a pseudo-random order. Each trial was manually started by the experimenter, using Max MSP software (https://cycling74.com/). For deaf participants, only the two vibrotactile conditions were presented, counterbalanced in order between participants. For hearing participants, the two vibrotactile conditions were presented first, counterbalanced in order between participants, followed by the auditory condition. Therefore, deaf and hearing participants experienced a total of 12 and 18 testing trials, respectively. After testing was complete, participants filled out a questionnaire regarding their dance and musical experience. Deaf participants were additionally interviewed about their deafness history and language experience.

### Data analysis

**Figure 1:**
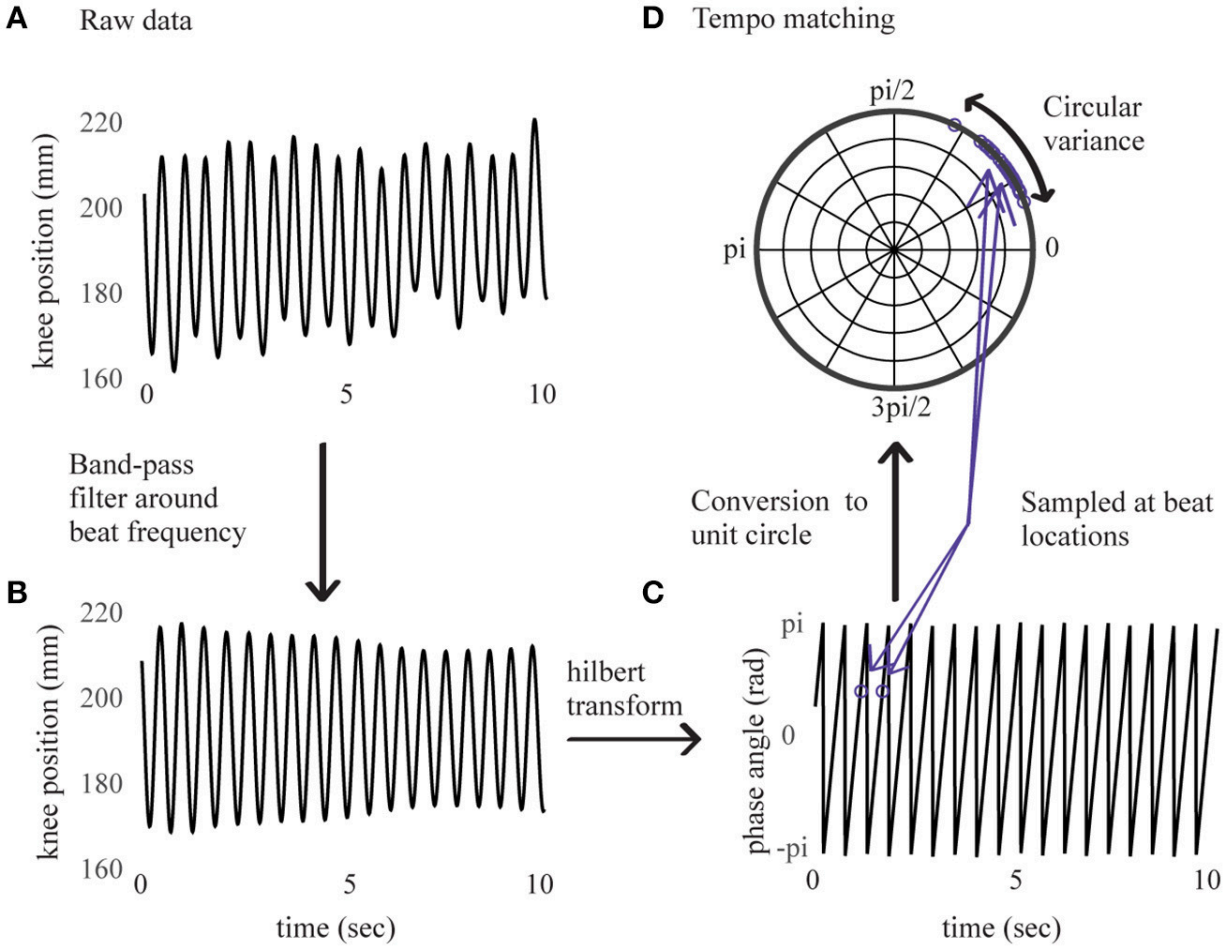
Right knee's displacement in direction of maximal amplitude for an example sample of 10 s. Before band-pass filtering in **(A)**, after band-pass filtering in **(B)**, corresponding instantaneous phase values from Hilbert transform in **(C)**, sampled at beat locations and converted to the unit circle in **(D)**.

Continuous movement data were analyzed using the Motion Capture (MoCap) Toolbox (Toiviainen and Burger, 2011) in Matlab. We visually checked the full-body recordings of each participant to ensure that the movement performed was consistent with the experiment instructions. Since this movement was constrained to an up-down bouncing motion, we expected that the trajectories of the moving markers would be correlated. Thus, we analyzed data from the right knee because knee displacement is highly constrained by the bouncing movement. For each trial, displacement data of the marker on the right knee was extracted. The first step in the analysis was to determine whether the movement was phase-locked to the stimulus beat, indicating whether the bouncing tempo consistently matched the stimulus tempo. To this aim, we performed a procedure similar to one described in Phillips-Silver et al. (2011). Data were first band-pass filtered around a center frequency corresponding to the stimulus' beat frequency and a bandwidth of 20% of the beat frequency (Figure 1). The component of maximal movement amplitude was then selected. For all trials, it corresponded to the direction of the axis parallel to the floor and perpendicular to the wall faced by the participant (horizontal anteroposterior direction).

The signal's instantaneous phase was subsequently extracted using a Hilbert transform (Figure 1) and sampled at time locations corresponding to the beats in the stimulus. Discrete phase values corresponding to the first 10 beats were removed (i.e., ~5 s of data) to leave participants some time to find the beat, and the analysis was performed on values corresponding to the next 115 beats. Phase relations between movement and stimulus were then converted to vectors on the unit circle and subjected to a Rayleigh test. A significant (*p* < 0.001) result for the Rayleigh test means that the vectors are centered at a preferred direction (the mean vector), thus indicating that the movement was phase-locked in relation to the stimulus' beat frequency. A non-significant result for the Rayleigh test means that the distribution of vectors on the circle cannot be distinguished from a uniform distribution, thus indicating that the relation between movement and beat frequency is at chance.

For each phase-locked trial, we used the circular variance (Batschelet, 1981) of the vectors to characterize the quality of synchronization. The circular variance is a measure of angular dispersion, indicating how well the movement and stimulus' phases were consistently aligned. This score is bounded by zero (constant phase relation), with lower values corresponding to higher consistency between the movement and stimulus phases. Distribution of the circular variance corresponded to a lognormal function. To normalize the distribution, the inverse logarithm of the circular variance was taken as a measure of synchronization consistency, higher values representing higher performance, and will henceforth be referred to as “synchronization consistency” (SC) score.

## Results

A significant Rayleigh test (i.e., the relation between movement and beat frequency is above chance level) was obtained for the vast majority of the data (341 of 366 trials in total). Because our goal was to study the quality of successful synchronization (Iversen et al., 2015), we excluded the trials that were not phase-locked (five trials out of 84 for the deaf group, and 10 trials out of 270 for the hearing group). Additionally, one participant in the deaf group failed to phase-lock bounces for more than half of the trials, demonstrating a failure to synchronize reliably. All of this participant's data were excluded from subsequent analyses. One participant from the hearing group had extremely poor synchronization consistency (SC) scores in the music condition (mean score inferior to two *SD* below the mean of the group) and was thus excluded from the final sample. Note that individuals with poor bouncing synchronization to music are not unusual (Tranchant et al., 2016). The final sample included seven deaf and 14 hearing participants (see Supplementary Materials for performance of excluded participants).

Statistical comparisons were performed using the ezANOVA function from the ez package in R (https://cran.r-project.org/web/packages/ez/index.html). For each participant, each stimulus presentation condition, and each tempo, the average SC score of the two trials was used in the following statistical comparisons.

### Synchronization to vibrotactile and auditory music in hearing group

**Figure 2:**
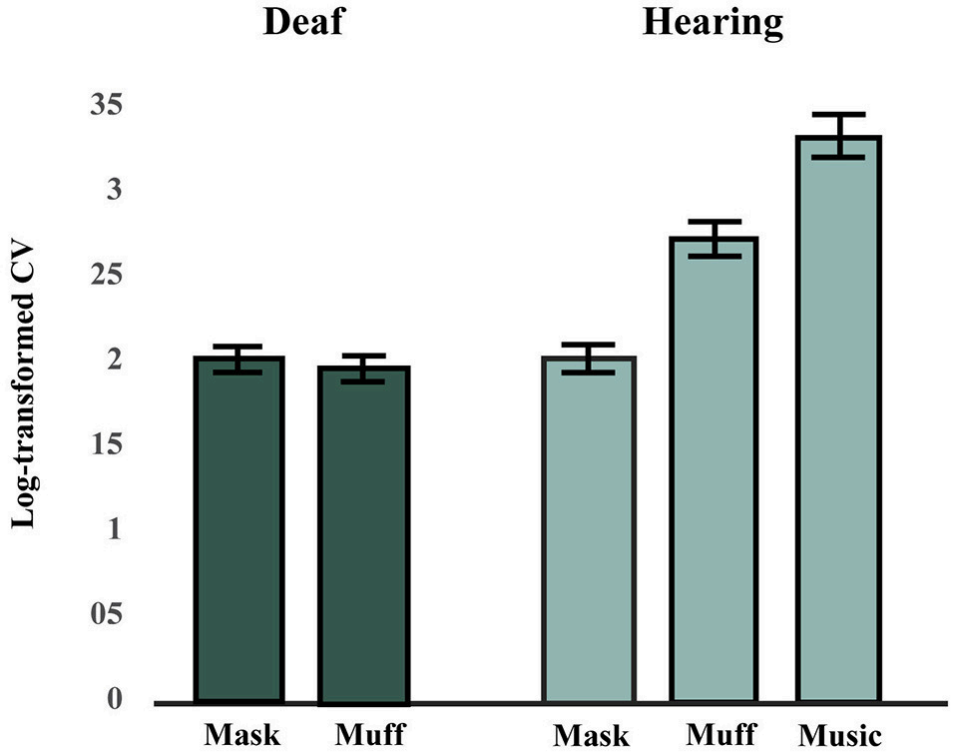
Synchronization consistency (SC) scores in Hearing and Deaf groups (averaged across tempi). A higher synchronization consistency (SC) score indicates better performance. The hearing group performed best in the music condition, followed by the muff and mask conditions. The deaf group performed worse in the vibrotactile conditions (mask and muff) than the hearing group in the muff condition, but similarly to them in the mask condition.

The effects of Stimulus Modality (mask, muff, auditory) and Tempo (110, 115, or 120 BPM) on SC scores in the hearing group were tested using a factorial repeated-measures ANOVA. There was a significant main effect of Stimulus Modality, *F*_(2, 26)_ = 27.11, *p* < 0.001, $\eta_{p}^{2}$ = 0.68. There was no main effect of Tempo, *F*_(2, 26)_ = 0.55, *p* = 0.58, $\eta_{p}^{2}$ = 0.0407, and no interaction effect between Stimulus Modality and Tempo, *F*_(4, 52)_ = 0.72, *p* = 0.58, $\eta_{p}^{2}$ = 0.0525. *Post-hoc* comparisons (Bonferroni-Holm *p*-value adjustment method) for the main effect of Stimulus Modality revealed that all conditions significantly differed from each other, all *p* < 0.001. SC scores were highest in the auditory condition and lowest in the mask condition, see Figure 2.

### Synchronization to vibrotactile music in deaf group

The effects of Stimulus Modality (mask, muff) and Tempo (110, 115, or 120 BPM) on SC scores in the deaf group were tested using a factorial repeated-measures ANOVA. There was no difference between the two conditions for Stimulus Modality, *F*_(1, 6)_ = 0.04, *p* = 0.85, $\eta_{p}^{2}$ = 0.0061, no effect of Tempo, *F*_(2, 12)_ = 1.91, *p* = 0.19, $\eta_{p}^{2}$ = 0.24, and no interaction effect between these two factors, *F*_(2, 12)_ = 0.71, *p* = 0.51, $\eta_{p}^{2}$ = 0.11, see Figure 2.

### Comparison between hearing and deaf groups

Although the deaf participants completed both vibrotactile conditions (mask and muff), we anticipated that they would show no difference between these conditions, since their deafness precluded them from experiencing potential residual noise of the vibrating platform, potential noise from bone conduction of vibrations, and the noise of the masking. Consistent with this hypothesis, there was no significant difference between the mask and muff conditions in the deaf group. As such, these conditions were averaged and compared to the vibrotactile with masking and with earmuffs conditions in the hearing group. Data were also averaged for tempo as no effect of that factor was found in either group. There was no difference between groups for the mask condition, *t*_(19)_ = 0.17, *p* = 0.87, but there was a significant difference between groups for the muff condition, *t*_(19)_ = 2.55, *p* = 0.020.

## Discussion

Our results indicate that both hearing and deaf individuals can synchronize a bouncing motion to a vibrotactile beat. This performance is consistent with results from previous research showing the effectiveness of the somatosensory system for conveying beat information in hearing people (Brochard et al., 2008; Giordano and Wanderley, 2015; Ammirante et al., 2016). Our finding extends previous work into a dance context, where (1) The vibrotactile stimuli mimicked those felt through a floor in a dance club, and (2) The response consisted of a whole-body movement, reflective of a basic dance behavior. Since deaf people were also capable of this behavior, our result is consistent with other research that suggests that deafness does not impair beat synchronization through non-auditory sensory modalities (Iversen et al., 2015). This conclusion is also supported by research in previously-deaf, cochlear-implant users, who show no deficit in beat synchronization to a metrical beat in music (Phillips-Silver et al., 2015).

Both the deaf and hearing groups synchronized to a vibrotactile beat less accurately than the hearing group did to an auditory beat. This enhanced behavior for the auditory condition may reflect superiority for this modality in beat synchronization, which is consistent with earlier research (Chen et al., 2002; Repp and Penel, 2002, 2004; Patel et al., 2005; Elliott et al., 2010; Ammirante et al., 2016). Alternately, our finding may reflect differences in the beat salience between the auditory and vibrotactile conditions. Since our goal was to reproduce an ecologically relevant dance experiment, our vibrotactile stimuli mimicked the response properties of a wooden floor and thus contained mainly low frequency information (see Supplementary Materials). The stimuli in the auditory conditions, by contrast, included higher frequency information, which conveyed additional rhythms that may have helped with synchronization (see Supplementary Materials). As well, the stimuli in the auditory condition may have been clearer than the vibrotactile conditions: In the vibrotactile conditions, the amplitude of the stimuli was limited to a level that would not produce sound that was audible to the participant through airwaves or bone conduction. Although all participants reported that they were able to easily feel the stimuli in the vibrotactile conditions, it is still possible that the auditory condition was more easily perceived. Similarly, we also speculate that the auditory and vibrotactile conditions may have varied in the sharpness of the onset of the beat, due to differences in the conduction properties of the different modalities (wooden platform for the vibration conditions vs. air for the auditory condition).

For the vibrotactile conditions, hearing people performed better for vibrations when wearing earmuffs as compared to when listening to auditory masking. We included both of these conditions in order to control for two possibilities. First, the use of auditory masking ensured that hearing people could not “hear” the vibrations, either through airwaves generated by the platform or through bone conduction through the body. However, since the masking introduced an extra stimulus to the hearing group that could not be replicated in the deaf group, we included a second condition, in which we masked only the sounds generated by the vibrations that traveled through airwaves, with earmuffs. To make sure that all conditions were equated as much as possible between groups, the deaf participants also experienced both vibrotactile conditions. No difference was predicted between the mask and muff conditions in the deaf group, since their profound hearing loss should block all auditory stimulation, and indeed we found no differences in behavior for these conditions in this group.

There are two possible explanations for the difference between the vibrotactile conditions in the hearing group. First, there may have been an advantage in the muff condition, as compared to the mask condition, because of incomplete blockage of auditory information due to bone conduction of the vibrations. Although all participants reported that they could not hear the vibrations with the earmuffs, we also noted anecdotally that some participants reported that it was difficult to determine whether they both felt and heard the vibrations, vs. only felt them, which may have influenced their self-reports. Second, the extra sensory information provided by the auditory masking stimulation may have interfered with performance in this condition. In line with this possibility, much research has documented the interference of somatosensation by auditory distraction (for a review, see Kitagawa and Spence, 2006). We recommend that these possible confounds are taken into consideration for future research. For example, implementing a systematic measure of participants' detection thresholds of vibrations through bone conduction may help account for the potential role of this factor when earmuffs are used. Likewise, including an additional auditory condition with vibrotactile noise may help control for the potential role of distraction by the masker.

We found no evidence to support our prediction that deaf people can synchronize better than hearing to a vibrotactile beat. In an attempt to capture behavior that could be acquired though a typical life experience of social dancing, we tested individuals who reported an enjoyment of dancing but no extensive training. Within this sample, we identified one hearing and two deaf participants who were unable to synchronize to the beat. Upon reviewing the full-body motion capture recordings for these excluded participants, we observed that both the deaf and hearing were able to produce a continuous and regular bouncing movement that was qualitatively indistinguishable from other participants. Thus, we presume that their difficulty lies in synchronization with the sensory stimulus. Previous research on beat synchronization in a large sample of typical adults identified 14% of participants that could not maintain auditory-motor synchronization (Tranchant et al., 2016). The outlier participants in the current study may belong to this population, which would be an indication that this difficulty with sensorimotor synchronization is not dependent on auditory experience. Alternately, the two excluded deaf participants could be evidence of an effect of auditory deprivation on beat synchronization. Given that a hearing participant was also excluded, our data do not provide strong support for this possibility, but it may be assessed in future research with a larger sample of deaf participants, and a more thorough assessment of motor and cognitive abilities to rule out other potential confounding factors.

Our final sample consisted of 14 hearing and seven deaf participants, who were able to synchronize without having had extensive training during their lifetime. With this homogeneous level of experience, we could not measure the potential influence of experience on beat synchronization performance. This behavior may be susceptible to the effects of long-term training, as is suggested by the improved beat synchronization in musicians as compared to non-musicians (Matthews et al., 2016). Whether or not deafness increases the potential of such training effects remains a topic for future research.

The absence of a difference between deaf and hearing people does not support the hypothesis that auditory deprivation leads to compensatory enhancement of the somatosensory system to support dancing. This is despite evidence that deaf people have enhanced sensitivity to vibrotactile stimuli (Levanen and Hamdorf, 2001) and may be an indication that enhancements to somatosensory processing in the deaf are limited to specific behaviors, which exclude beat synchronization. A similar selectivity for enhancements has been found in the visual domain, where deaf people are better than hearing at detecting visual motion (Shiell et al., 2014), but not at determining its direction (Bosworth and Dobkins, 1999), or detecting changes in velocity (Brozinsky and Bavelier, 2004). As an alternative to enhanced beat synchronization, compensatory vibrotactile sensitivity in deaf people (Levanen and Hamdorf, 2001) may support improvements to sensory-driven attention. This interpretation has been proposed for understanding enhancements to the visual system after deafness (Shiell and Zatorre, 2016; Shiell et al., 2016, see discussion in Shiell, 2015). The reasoning for this interpretation follows that the auditory modality has access to information about the full surroundings, and not just the space that falls within the line of vision or the reach of touch. As such, audition is ideal for monitoring the environment outside of the current focus of attention and detecting potentially relevant stimuli so that attention can be reoriented toward them. In the case of auditory deprivation, the remaining sensory systems may compensate for the missing sense through enhanced interactions with the neural system for sensory attentional reorienting. Following this reasoning, we predict that deaf people may be more distracted by extraneous vibrotactile information than hearing people, which could be tested in future research with the vibrating platform of the current experiment.

In previous research, deaf people showed more auditory cortex activity than hearing people in response to a vibrotactile stimulus, indicative of cross-modal reorganization of the auditory cortex (Levanen et al., 1998; Auer et al., 2007). Whether or not this cross-modal activity is relevant for beat synchronization remains to be seen. It may be that vibrotactile beat synchronization in deaf people is supported by different neural circuits than that of hearing people, despite similar behavioral outcomes in both groups. This possibility mirrors an example of cross-modal reorganization in the blind population, where performance on a tactile task was similar to that observed in sighted people, despite the fact that the task elicited increased activity in the visual cortex of the blind as compared to the sighted (Ptito et al., 2005).

The absence of an enhancement of vibrotactile beat synchronization in deaf people may indirectly support the importance of the visual system in this process. Potentially, deaf dancers follow the beat using cues from the movements of other dancers and musicians. This proposal is consistent with evidence that deaf people are better than hearing people at synchronizing a finger tapping motion to a visual flash stimulus (Iversen et al., 2015). This visual advantage for the deaf may extend to the whole-body movements studied here. Unlike this previous work on visual beat synchronization, in our experiment we used a naturalistic musical stimulus and continuous movements, which may be different than the discrete stimulus and movement used in earlier research (Iversen et al., 2015). Further investigation into differences between discrete and continuous vibrotactile beats may be a topic of interest for future research. In any case, the ability of the deaf group to synchronize to a vibrotactile beat validates the use of vibrotactile music technology for people with hearing impairments (e.g., Karam et al., 2009; Baijal et al., 2012), and this finding may help guide future developments in this field, particularly with regards to interventions for social dancing.

In conclusion, our findings support that both deaf and hearing people can synchronize to music-induced vibrations. This is in line with the fact that deaf people often participate in social dancing (Darrow, 1993) and provides evidence that vibrotactile beat information may guide this behavior. Overall, these results speak to the universality of dance behavior across sensory modalities and hearing experiences.

## Author contributions

PT, MS, MG, RZ, and IP conceived and designed the work. PT, MS, and AN acquired the data, with technical support from MG. PT, MS, and AN reviewed the data. PT analyzed the data in consultation with MS, RZ, and IP. PT, MS, and AN wrote the manuscript, with revisions and/or approval from MG, RZ, and IP.

### Conflict of interest statement

The authors declare that the research was conducted in the absence of any commercial or financial relationships that could be construed as a potential conflict of interest.
