# Supplementary material for: Feeling the Beat: Bouncing Synchronization to Vibrotactile Music in Hearing and Early Deaf People
Source: Front Neurosci. 2017 Sep 12;11:507. doi: 10.3389/fnins.2017.00507 (PMC5601036; doi:10.3389/fnins.2017.00507)
Supplement: Supplementary file 1 [file DataSheet1.PDF]

Supplementary Figures

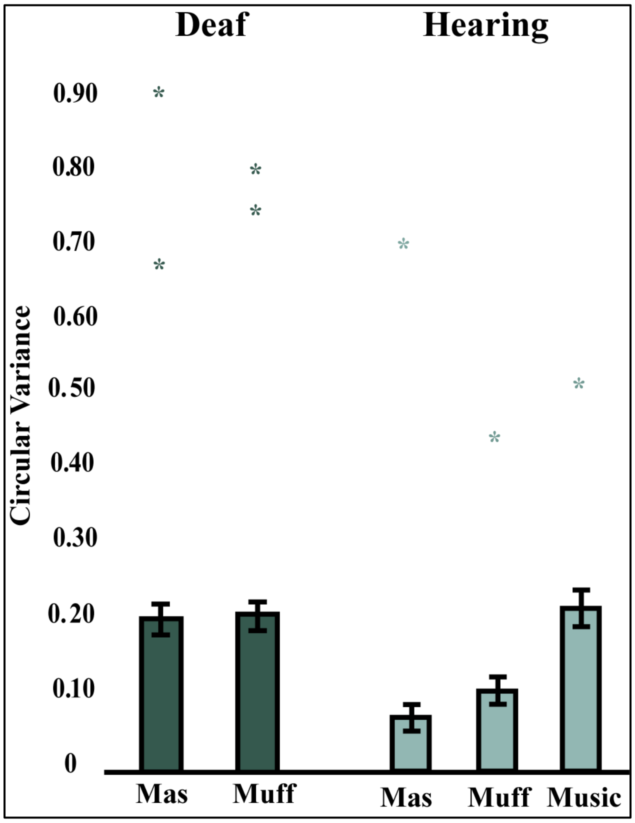

Non-transformed circular variance in each condition with data from excluded participants denoted as asterisks.

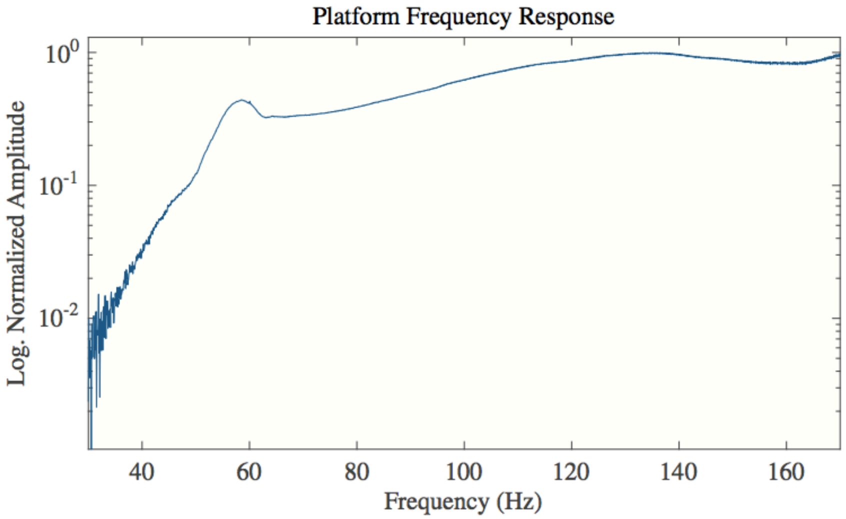
Platform Frequency Response
Log. Normalized Amplitude
10^0
10^{-1}
10^{-2}
40
60
80
100
120
140
160
Frequency (Hz)

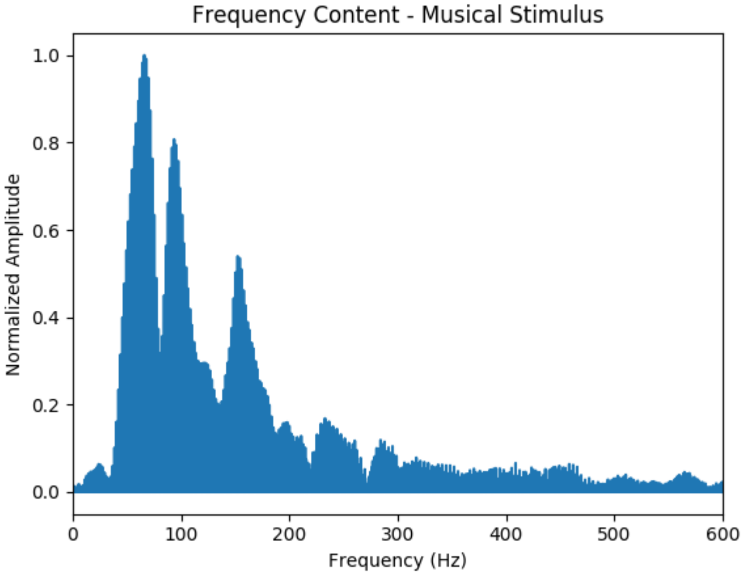
Frequency Content - Musical Stimulus
Normalized Amplitude
1.0
0.8
0.6
0.4
0.2
0.0
0
100
200
300
400
500
600
Frequency (Hz)

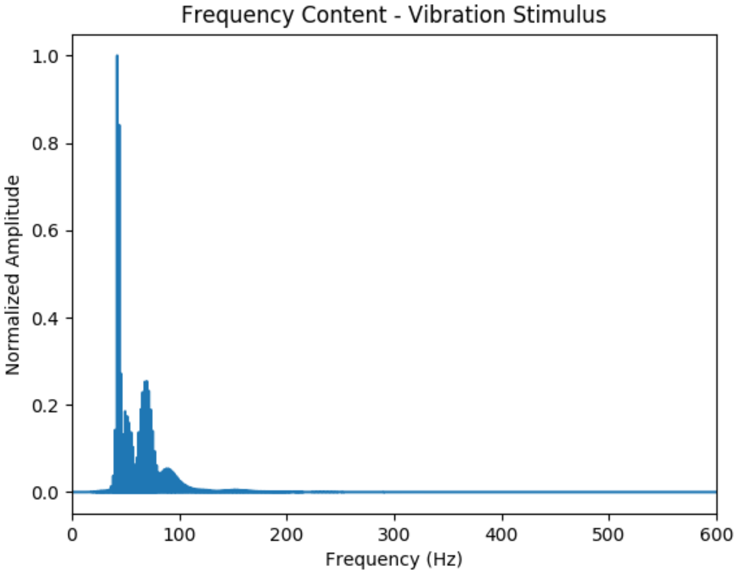
Frequency Content - Vibration Stimulus
Normalized Amplitude
1.0
0.8
0.6
0.4
0.2
0.0
0
100
200
300
400
500
600
Frequency (Hz)
